# Psychometric properties of the Greek version of the NEI-VFQ 25

**DOI:** 10.1186/1471-2415-8-4

**Published:** 2008-03-06

**Authors:** Georgios Labiris, Andreas Katsanos, Michael Fanariotis, Theodora Tsirouki, Maria Pefkianaki, Dimitrios Chatzoulis, Evangelia Tsironi

**Affiliations:** 1 Ophthalmology Clinic, University of Thessaly, Larissa, Greece; 2 Intermedico Network, Athens, Greece

## Abstract

**Background:**

To evaluate the reliability and construct validity of a Greek version of the NEI-VFQ-25 in patients with chronic ophthalmic diseases.

**Methods:**

We developed the Greek version of the instrument using forward and backward translation. One hundred-eighty-six patients responded to the questionnaire. To examine reliability, Cronbach's alpha for each subscale was used as an index of internal consistency. Test-retest reliability was evaluated with intraclass correlation coefficients. Regarding construct validity, both convergent and discriminant validities were calculated by means of multi-trait analysis. Rasch analysis was used to estimate the visual ability required by each item for a particular response, and each patient's visual ability. Correspondingly, instrument validity was evaluated by estimating the distribution of residuals for item and subject measures.

**Results:**

Four patient groups were studied, each including participants with a single cause of visual impairment. Group 1 consisted of 84 glaucoma subjects. Group 2 included 30 subjects with age-related macular degeneration (ARMD); group 3 included 25 subjects with dry-eye syndrome, whereas group 4 included 18 cataract patients. Twenty-nine healthy individuals comprised the control group. NEI-VFQ scores (mean ± SD) for the glaucoma, ARMD, dry-eye, cataract and control groups were: 76.9 ± 20.2, 70.9 ± 20.2, 81.6 ± 16.5, 73.5 ± 24.0 and 93.7 ± 8.9 respectively. Item analysis revealed no significant data skewing. Cronbach's alpha ranged from 0.678 to 0.926, with most subscales having high internal consistency. Intraclass correlation coefficient ranged from 0.717 to 0.910 for all subscales. All items passed the convergent and discriminant validity tests. Strong correlations were detected between visual acuity and "general vision", "distant activities" and "near activities" subscales. Significant correlations were also detected between visual field deficits and the "peripheral vision" and "general vision" subscales. Rasch analysis revealed potential weaknesses of the instrument that are associated with the assumptions of the model itself. Specifically, low precision of the "agreement" items was detected in the estimation of visual ability. Twenty-three percent of the subjects had fit statistics that fell outside the tolerance box.

**Conclusion:**

Although traditional validation methods indicated that the Greek version of the NEI-VFQ-25 is a valid and reliable instrument for VS-QoL assessment, Rasch analysis detected significant misfits to the model, especially of the "agreement" items. This means that results of the corresponding subscales should be interpreted with extreme caution.

## Background

The impact of ophthalmic diseases on quality of life (QoL) has been documented in a series of studies [1-4]. Numerous instruments have been developed: general, vision-specific, or disease specific ones (i.e. glaucoma-specific QoL instruments) that evaluate patients' subjective perceptions regarding QoL [5-8]. In the majority of QoL studies, an association is attempted between objective clinical indices that quantify functional status and a series of quality dimensions that purportedly reflect QoL. However, vision-specific QoL (VS-QoL) studies in Greek populations are fragmented. In fact, apart from the prevalent general QoL questionnaires, none of the vision-specific or ophthalmic-disease-specific instruments has been validated in Greek populations [9].

The NEI-VFQ 25 (National Eye Institute visual function questionnaire) is a widely used vision-specific instrument for the assessment of VS-QoL. The instrument was originally developed by the National Eye Institute mainly for the English-speaking North American populations [10]. NEI-VFQ 25 is a short form of the original 51-item version [11]. Reliability and validity of the English version of the NEI-VFQ 25 have been evaluated and found comparable to the 51-item version. NEI-VFQ 25 measures the following vision-depended functions: General health, general vision, ocular pain, near activities, distant activities, social functioning, mental health, role difficulties, dependency, driving, color vision and peripheral vision. A 0–100 point scale is used for subscale scores. A score of 100 indicates the best possible score, while 0 indicates the worse possible score. With slight modifications, the original NEI-VFQ 25 has been translated and validated for other populations as well [12-15]. The original NEI-VFQ 25 has been used as an index of VS-QoL for a series of diseases that exert variable impact on visual functional capacity, including glaucoma [16], age-related macular degeneration [17], diabetic eye disease [18], dry eye syndrome [19], blepharospasm [20], and retinitis pigmentosa [21]. Moreover, the instrument has been used as an index of the impact of therapeutic interventions and rehabilitation programs on VS-QoL [22-24].

The aim of our study was the evaluation of the reliability and validity of NEI-VFQ 25 in a sample of native Greek subjects with a series of common ophthalmic diseases.

## Methods

### Instrument development

The study adhered to the tenets of the Declaration of Helsinki and approval was granted by the Bioethics Committee of the University of Thessaly. All participants provided written informed consent.

For this study we developed a Greek version of the NEI-VFQ 25. The original NEI-VFQ 25 is a VS-QoL instrument that has been used in numerous studies. The concepts investigated by the items have been extensively analyzed both during the development of the original version and during the validation of the instrument in other languages. Forward translation was done by native Greek ophthalmologists with solid command of the English language. Two independent bilingual speakers performed the backward translation. Proper adaptation of the questionnaire to the experience of Greek patients mandated slight modification of three questions. Thus, item "13" [How much difficulty do you have visiting people at their homes, at parties, or in restaurants?] was translated as: [How much difficulty do you have visiting people at their homes or outdoors, to restaurants, cafes, or in church?]. Accordingly, item "A7" [Because of your eyesight, how much difficulty do you have taking part in active sports or other outdoor activities that you enjoy (like golf, bowling, jogging, or walking)?] was translated as: [Because of your eyesight, how much difficulty do you have taking part in active sports or other outdoor activities that you enjoy (going long walks or jogging)?]. On the other hand, item "9" [Because of your eyesight, how much difficulty do you have going down steps, stairs, or curbs in dim light or at night?] was modified as follows: [Because of your eyesight, how much difficulty do you have going down stairs, or curbs in dim light or at night?], since the Greek words for "stairs" and "steps" are almost identical. The Greek version of the instrument was pilot-tested in a sample of ten ophthalmic patients who visited the outpatient service of our clinic for their annual check-up. The results of the pilot-testing indicated that the instrument was well accepted, since it was short in duration (about 10 minutes) and all items were easy to understand.

It should be mentioned that the NEI-VFQ 25 has already been translated into Greek (Laboratory of Experimental Ophthalmology of Aristotle University, Thessaloniki, Greece, 2000) [25]. However, to our knowledge, it has not been validated in native populations. In addition to the aforementioned modifications required by the proper adaptation of the instrument in Greek language, a series of minor differences in the expression of the items suggested a thorough revision of the translation process. Nevertheless, our translation of the NEI-VFQ 25 was almost identical to the one produced by the Laboratory of Experimental Ophthalmology of the Aristotle University.

### Study Design

Evaluation of the reliability and validity of the Greek version of the NEI-VFQ 25 questionnaire was conducted by the University Eye clinic, Larissa, Greece, between August and November 2006. The University Hospital of Larissa is an 800-bed General Hospital, located at the center of mainland Greece, offering tertiary healthcare to more than one million beneficiaries. The University Eye Clinic provides integrated ophthalmic services to patients from four counties in mainland Greece and one on the islands. Two hundred fifty patients were recruited for the study. Of them, 186 successfully responded to the questionnaire, including the test-retest module (response rate: 74.4%). The patients were randomly selected from the local national health system hospitals of the corresponding counties.

### Subject groups

Subject group 1 consisted of a random sample of 84 known glaucoma patients (38 males/46 females). All subjects had been diagnosed with primary open angle glaucoma (POAG), pseudoexfoliation glaucoma (PXG), pigment glaucoma (PG) or normal tension glaucoma (NTG) at least two years prior to the study, and demonstrated adequate compliance. Glaucoma patients had bilateral typical glaucomatous visual field defects on Humphrey 24-2 tests, untreated intraocular pressure higher than 22 mmHg (except for the NTG patients) and either retinal nerve fibre layer defects compatible with glaucoma on OCT, or optic nerve heads with a typical glaucomatous appearance. Subject group 2 consisted of a random sample of 30 age-related macular degeneration (ARMD) patients (14 males/16 females). To be eligible for the ARMD group, each subject should have variable bilateral involvement presenting either with geographic atrophy involving the fovea, choroidal neovascular membrane, or pigment epithelium defects. Subject group 3 consisted of a random sample of 25 patients (1 male/24 females) diagnosed with dry-eye syndrome. These had been diagnosed according to the European classification criteria [26] and presented Schirmer score <5 mm at 5 minutes with abnormal Ocular Surface Discomfort Index scores. Subject group 4 consisted of a random sample of 18 cataract patients (5 males/13 females). Cataract subjects were recruited from the waiting lists for cataract extraction. Among the eligibility criteria for all subjects was the necessity for a single reason of visual impairment that classified them accordingly to the abovementioned groups. Subsequently, patients presenting more than one causes of visual impairment (e.g. POAG and ARMD) were excluded. Subjects with serious mental and/or major systemic diseases were also excluded. The control group consisted of a random sample of 29 adults (6 males/23 females) who visited the outpatient office for their annual ophthalmic checkup and had no apparent cause of visual impairment.

### Data collection

Subjects responded to the self-administered Greek version of the NEI-VFQ 25 in the presence of an independent researcher who had no direct involvement in the provision of care. All questionnaires were completed prior to the clinical examination. Clinical and demographic data regarding the participants was retrieved from their medical records. Proxy responses (i.e. from family members) were excluded.

### Descriptive analysis and item analysis

Data from the different subject groups were used for the item analysis. Missing values were estimated for each item. Large ceiling or floor effects were evaluated as well.

### Reliability

Reliability analysis was done by Cronbach's alpha estimation as an index of internal consistency for each subscale [27]. A time window of three weeks between two consecutive surveys (20 ± 5 days) was used for the assessment of test-retest reliability. Quantification of test-retest reliability was done using intraclass correlation coefficients.

### Construct validity

Construct validity was evaluated by means of multi-trait analysis according to Campbell and Fiske [28]. It is known that convergent and discriminant validities are subtypes of construct validity. According to the multitrait-multimethod matrix, convergent validity is the degree to which concepts that should be related theoretically are interrelated in reality, while discriminant validity is the degree to which concepts that should *not *be related theoretically, are in fact *not *interrelated in reality. In brief, each item was hypothesized to belong to only one multi-item subscale and correlations between the score on that item and the scores on all the subscales were computed. Then, for each item, if the correlation between the score on that item and the score on the subscale to which that item belongs is 0.4 or higher, that item is validated in terms of convergent validity. On the other hand, each item was validated for discriminant validity if the correlation between the score of the item and the score on the subscale where it belongs is greater than all the correlations among the score of the item and the remaining subscales.

### Rasch analysis

Besides the traditional methods, the psychometric properties of the Greek NEI-VFQ 25 were also evaluated by Rasch analysis. The item response theory has been described in earlier studies [29,30]. Rasch models have been used in the validation of a series of vision-specific and disease-specific QoL instruments [31,32] as well as the assessment of therapeutic interventions on QoL [33]. In brief, item response models attempt to estimate the values of latent variables on an interval scale from item scores that form an ordinal scale. "Visual ability" is the variable of interest regarding the assessment of visual function by means of the item response theory. Each patient is supposed to have a unique visual ability that determines the difficulty in performing certain daily tasks. In fact, each activity requires a certain visual capacity in order to be performed with ease.

The tests of construct validity measure the fit of the person measures to the model, and the correlations of person- and item parameter values with other variables, compared with expected correlations. The tests of content validity measure the fit of individual items to the model, the estimation errors of item parameter values, and the spacing and range of item parameter values, relative to the distribution of person parameter values.

### Statistical analysis

Statistical analyses were performed with SPSS^® ^version 13 for Windows (SPSS Inc, Chicago, IL). Rasch analysis was performed with the Winsteps programme (Linacre, 2007).

## Results

Regarding glaucoma subjects, 43 patients had primary open angle glaucoma (POAG), 10 had pseudoexfoliation glaucoma (PXG), 16 had normal tension glaucoma (NTG) and 15 subjects had ocular hypertension (OH). The MD ranged from -1.9 dB to -7.1 dB in Humphrey 24-2 threshold testing (mean: -3.2 dB). Regarding ARMD subjects, 28 patients presented variable degrees of bilateral geographical atrophy, while 2 patients presented exudative changes in one eye. Regarding cataract subjects, mean LOCS III score for nuclear opalescence was 3.1 and LOCS III score for nuclear color was 3.3. Regarding the dry-eye syndrome group, all subjects were treated with artificial tears, 13 subjects reported that artificial tears treatment was effective, while 17 subjects demonstrated combined fluorescein score > 0.5. Demographics and clinical data for the participants are presented in Table 1.

**Table 1:** Demographics and clinical data

	**Glaucoma**	**ARMD**	**Cataract**	**Dry Eye**	**Control**
**Mean ± SD age (years)**	60.9 ± 11.7	65 ± 4.4	63 ± 7.3	59.1 ± 8.7	56 ± 8.9
**males/females**	38/46	14/16	5/13	1/24	6/23
**mean BCVA best (range)**	0.63 (0.5 to 0.8)	0.4 (0.67 to 0.32)	0.5 (0.63 to 0.32)	0.8 (0.67 to 1.0)	1
**mean BCVA worse (range)**	0.4 (0.33 to 0.63)	0.33 (0.25 to 0.40)	0.29 (0.16 to 0.4)	0.67 (0.5 to 0.8)	0.8 (1 to 0.67)
**VF MD (dB) (range)**	-3.2 (-7.1 to-1.9)	-	-	-	-
**0 medical comorbitities**	8	4	6	4	13
**1 medical comorbitities**	47	8	5	15	8
**≥2 medical comorbitities**	29	18	7	6	8

Mean subscale and overall NEI-VFQ scores for the different groups of subjects are presented in Table 2. Mean NEI-VFQ scores ranged from 81.6 ± 16.5 for the dry-eye patient group to 70.9 ± 20.2 for the ARMD group. Control subjects presented mean overall NEI-VFQ score of 93.7 ± 8.9.

**Table 2:** Index and subscale scores

	**Subject Group**									
**NEI-VFQ 25 subscales**	Glaucoma		ARMD		Dry-eye		Cataract		Control	
	
	Mean	SD	Mean	SD	Mean	SD	Mean	SD	Mean	SD

**General health**	55.3	20.3	57.6	17.1	45.9	17.2	57.8	19.7	80.3	12.2
**General vision**	69.6	15.5	56.6	10.9	75.1	15.2	60	17.4	90.7	12.9
**Ocular pain**	69.9	23.1	94.2	16	60.5	18.9	84	20.5	89.7	12.1
**Near activities**	78.7	18.7	58.6	23	86.2	16.3	65.6	26.9	96.1	9.7
**Distance activities**	82.2	19	74.4	17.7	90.2	13.9	74.7	18.5	96	7
**Vision specific social functioning**	89.2	19.4	89.1	12.8	94.1	9.7	84.3	22.9	99.4	3.1
**Vision specific mental health**	69.7	21.6	63.3	22.4	80.8	19.3	67.8	24.5	89.7	10.4
**Vision specific role difficulties**	74.3	21.7	59.8	24.4	86.5	18.7	68	29.3	94.8	12.1
**Vision specific dependency**	82.5	25.1	70.3	22.5	91.3	19.8	79.6	26.4	97.7	7
**Driving**	81.1	16.9	61.1	26.4	90	11.5	75	35.4	93.8	8
**Color vision**	88.3	19	86.7	23.4	89.3	22.1	86.1	17.6	100	0
**Peripheral vision**	82.4	21.4	79.2	25.5	89.6	15.4	79.4	28.3	95.7	11.7
**Overall Score**	76.9	20.2	70.9	20.2	81.6	16.5	73.5	24	93.7	8.9

Item analysis is presented in Table 3. The highest missing values were identified in the questions regarding "Driving" (missing percentages of 13.4% and 68.2% in items 15 and 16 respectively). On the other hand, significantly lower missing percentages were identified in the remaining items with higher values (7.6%) both in item 22 ("Have less control because of poor eyesight") and item 23 ("Rely on others because of poor eyesight"). Ceiling and floor values of the sample suggested that the data was not strongly skewed.

**Table 3:** Item analysis

**Item**	**Missing responses**		**Floor responses**		**Ceiling responses**	
	Number	%	Number	%	Number	%

**1. General Health: 5-level health rating**	1	0.5	14	8.9	6	3.8
**2. General Vision: 6-level general vision**	1	0.5	1	0.6	6	3.8
**3. Mental Health: Amount true: worry**	2	1.3	21	13.4	11	7.0
**4. Ocular Pain: Amount pain**	2	1.3	1	0.6	55	35.0
**5. Near Vision: Reading normal newsprint**	4	2.5	21	13.4	38	24.2
**6. Near Vision: See well up-close**	1	0.5	1	0.6	46	29.3
**7. Near Vision: Finding objects on crowded shelf**	1	0.5	5	3.2	88	56.1
**8. Distance Vision: Reading street signs**	2	1.3	6	3.8	64	40.8
**9. Distance Vision: Going down stairs at night**	2	1.3	2	1.3	61	38.9
**10. Peripheral Vision: Seeing objects off to side**	2	1.3	1	0.6	82	52.2
**11. Social Function: Seeing how people react**	2	1.3	2	1.3	99	63.1
**12. Color Vision: Difficulty matching clothes**	1	0.5	1	0.6	101	64.3
**13. Social Function: Visiting others**	1	0.5	4	2.5	114	72.6
**14. Distance Vision: Going out to movies/plays**	6	3.8	56	35.7	56	35.7
**15. Driving: Daylight familiar places**	21	13.4	84	53.5	52	33.1
**16. Driving: At night**	50	68.2	1	0.6	12	7.6
**17. Role Limitation: Accomplish less**	8	5.1	6	3.8	53	33.8
**18. Role Limitation: Limited in endurance**	10	6.4	6	3.8	53	33.8
**19. Ocular Pain: Amount time: pain**	8	5.1	1	0.6	73	46.5
**20. Dependency: Stay home most of time**	6	3.8	14	8.9	80	51.0
**21. Mental Health: Amount true: frustrated**	9	5.7	11	7.0	76	48.4
**22. Mental Health: Amount true: no control**	12	7.6	6	3.8	84	53.5
**23. Dependency: Rely too much on other's word**	12	7.6	4	2.5	92	58.6
**24. Dependency: Need much help from others**	10	6.4	6	3.8	89	56.7
**25. Mental Health: Amount true: embarrassment**	9	5.7	8	5.1	100	63.7

Evaluation of the reliability of the Greek version of the NEI-VFQ 25 is presented in Table 4. Cronbach's alpha ranged from 0.678 for the "Vision-specific social functioning" subscale to 0.926 for the "Vision specific role difficulties". Nevertheless, the majority of the subscales presented high internal consistency. Regarding test-retest reliability, intraclass correlation coefficient was higher than 0.7 in all subscales; it ranged from 0.717 for the "Color vision" to 0.910 for the "Vision-specific social functioning".

**Table 4:** Reliability and validity analysis

**Subscale**	Number of items	Intraclass correlation coefficients	Cronbach's alpha	Range of item-scale correlations	Convergent validity	Discriminant validity
**General Health**	1	0.841	NA	NA	NA	NA
**General Vision**	1	0.889	NA	NA	NA	NA
**Ocular pain**	2	0.803	0.851	0.893 – 0.903	100	100
**Near activities**	3	0.901	0.734	0.719 – 0.875	100	100
**Distance activities**	3	0.908	0.686	0.752 – 0.892	100	100
**Vision specific social functioning**	2	0.91	0.678	0.887 – 0.889	100	100
**Vision specific mental health**	4	0.823	0.786	0.596 – 0.837	100	100
**Vision specific role difficulties**	2	0.888	0.926	0.902 – 0.907	100	100
**Vision specific dependency**	3	0.826	0.848	0.835 – 0.899	100	100
**Driving**	3	0.871	0.757	0.575 – 0.953	100	100
**Color vision**	1	0.717	NA	NA	NA	NA
**Peripheral vision**	1	0.84	NA	NA	NA	NA

N.A.: Not applicable (needs two or more items).

Evaluation of the validity of the Greek version of NEI-VFQ 25 is presented in the multitrait-multimethod matrix (see Additional file 1) and Table 4. All items passed the convergent and discriminant validity tests.

The impact of visual acuity and visual field deficits on vision-specific quality of life is presented in Table 5. Strong Pearson correlations were detected between BCVA and the corresponding subscales that are associated with central vision (i.e. general vision and near activities). Correspondingly, strong Pearson correlations were detected between VF scores and "peripheral vision" and "general vision subscales".

**Table 5:** Pearson correlations of NEI VFQ 25 subscales with visual acuity and visual fields

		**BCVA worse**	**BCVA better**	**VF worse**	**VF better**
**General Health**	Pearson Correlation	-0.145	-0.078	-0.175	-0.145
	Sig. (2-tailed)	0.231	0.521	0.153	0.238
**General Vision**	Pearson Correlation	-0.279(*)	-0.251(*)	-0.444(**)	-0.407(**)
	Sig. (2-tailed)	0.019	0.035	0	0.001
**Ocular Pain**	Pearson Correlation	-0.082	-0.065	-0.1	-0.216
	Sig. (2-tailed)	0.499	0.589	0.415	0.075
**Near Activities**	Pearson Correlation	-0.331(**)	-0.337(**)	-0.361(**)	-0.411(**)
	Sig. (2-tailed)	0.005	0.004	0.002	0
**Distant Activities**	Pearson Correlation	-0.440(**)	-0.159	-0.260(*)	-0.346(**)
	Sig. (2-tailed)	0	0.186	0.031	0.004
**Social Functioning**	Pearson Correlation	-0.369(**)	-0.159	-0.227	-0.295(*)
	Sig. (2-tailed)	0.002	0.184	0.061	0.014
**Mental Health**	Pearson Correlation	-0.236(*)	-0.172	-0.021	-0.083
	Sig. (2-tailed)	0.047	0.15	0.862	0.498
**Role Difficulties**	Pearson Correlation	-0.218	-0.199	-0.204	-0.242
	Sig. (2-tailed)	0.074	0.103	0.1	0.05
**Dependency**	Pearson Correlation	-0.475(**)	-0.242	-0.174	-0.292(*)
	Sig. (2-tailed)	0	0.065	0.196	0.028
	N	59	59	57	57
**Driving**	Pearson Correlation	-0.239	-0.134	-0.371(*)	-0.041
	Sig. (2-tailed)	0.187	0.466	0.04	0.826
	N	32	32	31	31
**Color vision**	Pearson Correlation	-0.405(**)	-0.345(**)	-0.231	-0.288(*)
	Sig. (2-tailed)	0.001	0.004	0.062	0.019
**Peripheral vision**	Pearson Correlation	-0.22	-0.087	-0.334(**)	-0.336(**)
	Sig. (2-tailed)	0.07	0.478	0.006	0.005

**: Correlation is significant at the 0.01 level (2-tailed).

*: Correlation is significant at the 0.05 level (2-tailed).

**Figure 1:**
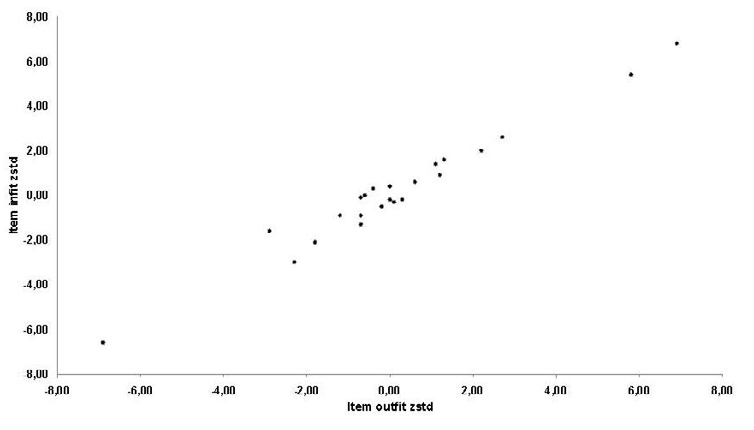
Infit and outfit values for the list of items.

**Figure 2:**
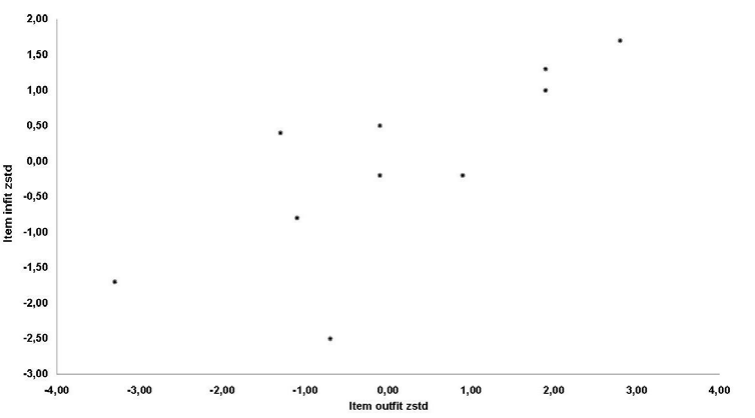
Infit and outfit values for the list of items – "agreement" items removed from model.

Regarding Rasch analysis, the normalized item fit statistics are presented in Table 6. The expected values are 0, with a tolerance of ± 2 deviation units. Positive values indicate that response residuals exceed expectations of the model, whereas negative values indicate that response residuals are less than the expectations of the model. Figure 1 illustrates the infit and outfit values for the list of items. Six items fell outside the tolerance box. The most misfitting items were the "overall eyesight" and the "overall health". In accordance with other researchers, gross misfits to the model were detected by the items that used agreement instead of difficulty rating scales. However, when the "agreement" items were removed from the model (table 7), the number of misfits decreased significantly (Figure 2).

**Table 6:** Estimates of item measures and fit statistics from Rasch analysis

**Item**	**Item Number**	***ρ***	**Standard Error**	**Infit zstd**	**Outfit zstd**
**Matching clothes**	12	-1.07	0.14	0.00	-0.20
**Finding something**	7	-0.92	0.13	0.10	-0.30
**See reaction**	11	-0.92	0.13	2.20	2.00
**Visit people**	13	-0.82	0.12	-0.70	-1.30
**Noticing objects**	10	-0.7	0.11	-0.70	-0.90
**Eye pain**	4	-0.42	0.10	-2.30	-3.00
**Going down steps**	9	-0.41	0.10	-1.80	-2.10
**Reading signs**	8	-0.4	0.10	-0.20	-0.50
**See up close**	6	-0.29	0.09	0.30	-0.20
**Overall eyesight**	2	-0.1	0.09	-6.90	-6.60
**Reading print**	5	-0.03	0.09	2.70	2.60
**Overall health**	1	0.15	0.08	5.80	5.40
**Worry about eyesight**	3	0.2	0.09	-1.20	-0.90
**See movies**	14	0.24	0.09	6.90	6.80
**Limited**	18	0.32	0.09	1.30	1.60
**Accomplish less**	17	0.32	0.09	-0.40	0.30
**Stay home**	20	0.44	0.09	1.10	1.40
**Feel frustrated**	21	0.48	0.09	1.20	0.90
**Less control**	22	0.69	0.10	0.00	0.40
**Need help**	24	0.71	0.10	-2.90	-1.60
**Eye discomfort**	19	0.72	0.10	-0.70	-0.10
**Rely too much**	23	0.88	0.11	0.60	0.60
**Embarrass myself**	25	0.94	0.11	-0.60	0.00

**Table 7:** Estimates of item measures and fit statistics from Rasch analysis (difficulty items only)

**Item**	**Item number**	***ρ***	**Standard Error**	**Infit zstd**	**Outfit zstd**
**Matching clothes**	12	-0.77	0.16	-0.70	-2.50
**Finding something**	7	-0.58	0.15	1.90	1.30
**See reaction**	11	-0.58	0.15	-0.10	0.50
**Visit people**	13	-0.43	0.14	-1.30	0.40
**Noticing objects**	10	-0.27	0.13	1.90	1.00
**Going down steps**	9	0.16	0.12	2.80	1.70
**Reading signs**	8	0.18	0.12	-0.10	-0.20
**See up-close**	6	0.34	0.11	0.90	-0.20
**Reading print**	5	0.74	0.11	-1.10	-0.80
**See movies**	14	1.2	0.11	-3.30	-1.70

**Figure 3:**
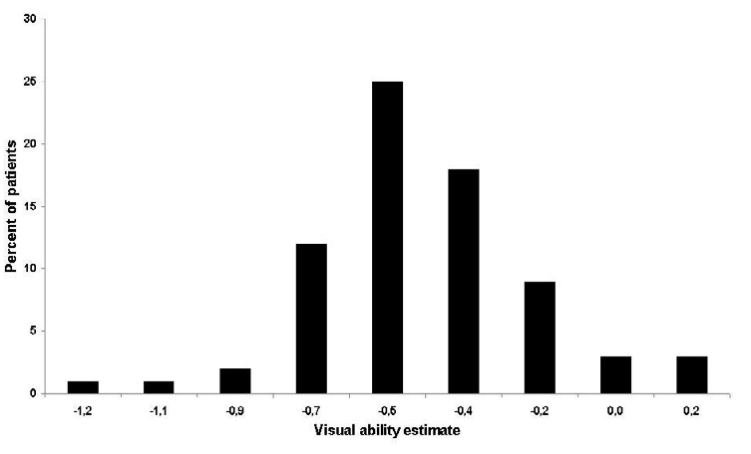
Distribution of visual ability measures.

**Figure 4:**
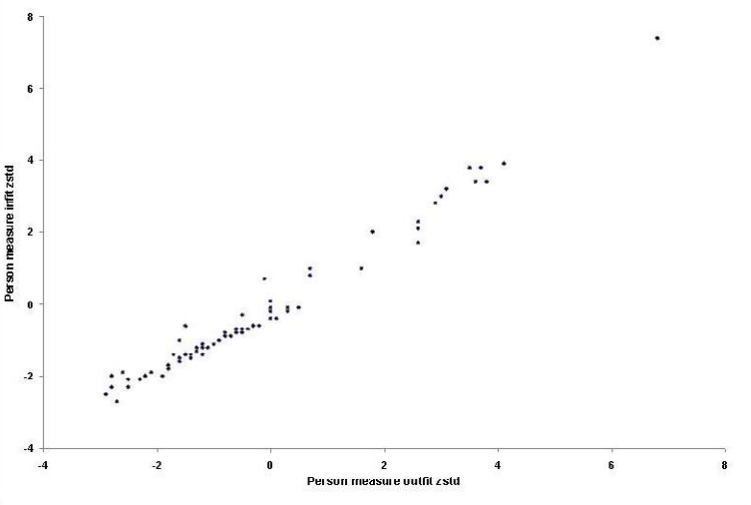
Distribution of the infit and outfit statistics for the estimates of visual ability.

Figure 3 illustrates the distribution of visual ability measures. Higher values indicate greater visual ability and less disability. On the other hand, lower values indicate lower visual ability and suggest that the subject is more disabled. The distributions of the infit and outfit statistics for the estimates of visual ability are presented in Figure 4. Twenty six percent of the patients had fit statistics that fell outside the ± 2 tolerance box.

## Discussion

The primary objective of our study was the evaluation of the reliability and construct validity of the NEI-VFQ 25 in native Greek populations with a series of common ophthalmic diseases. Proper adaptation of the instrument to the Greek norms mandated slight modification of some items and a thorough revision of the translation process that had already been performed by previous investigators. Minor modifications of some items were considered necessary during the translation and validation of the NEI-VFQ 25 in other populations, too [12,13,15].

Contrary to the original validation studies in North American populations, relatively high missing rates were encountered in some subscales. However, these missing rates were comparably lower than the ones encountered during the translation and validation of the instrument in other populations [13]. In fact, except for the "Driving" subscale, no significant missing rates were found. Thus, contrary to what Suzukamo and collaborators did, in our study no substitution was performed between the regular items with high missing rates and the corresponding optional ones with low missing rates within selected subscales [13]. On the other hand, high ceiling percentages were encountered in some items (i.e. "Social function: visiting others" and "Driving: driving at night"). However, no significant skewing of data was detected.

The subscales of the Greek version of NEI-VFQ 25 presented variable but adequate internal consistencies indicating high reliability of the instrument in the population studied. The lowest values of Cronbach's alpha were detected in "Social Functioning" (0.678) and "Distance Activities" (0.686) subscales, while the rest of the subscales presented significantly higher values. On the other hand, the time-window of three weeks between test and retest visits, ensured that no significant changes in the vision-related and/or systemic functional status of the subjects would take place. In fact, solid test-retest reliability was indicated by the high values of the intraclass correlation coefficients.

Regarding the construct validation of the questionnaire, none of the items failed either the convergent or the discriminant tests.

Strong correlations were detected between visual acuity of the subjects and the "general vision", "distant activities" and "near activities" subscales suggesting that the corresponding subscales were actually associated with central vision. Similar correlations between visual acuity and NEI-VFQ subscales have been detected by previous investigators during the validation of the instrument in other languages as well [14,15]. Moreover, in accordance to the concepts investigated by the corresponding items, the visual fields deficits of glaucoma subjects were associated with "peripheral vision" and "general vision" subscales. In fact, the results of our study regarding the impact of visual fields deficits on VS-QoL are similar to the ones by Ringsdorf and coworkers for the white, non-Hispanic population of their study [34].

On the other hand, item response analysis revealed potential weaknesses of the questionnaire that should be taken into consideration prior to the interpretation of results. These potential weaknesses are associated with the assumptions of the Rasch model. Among these assumptions are: a) only one variable is measured by the instrument, b) subjects' responses to the items depend only on visual ability, c) subjects' responses are probabilistic and conditional on the subjects' visual ability required to perform that activity with ease, d) the odds of performing an activity with ease increase monotonically with the difference between the subject's ability and the ability required to perform the activity with ease. The results of the study indicate that the items requiring agreement ratings presented low precision in the estimation of the latent variable, contrary to the items requiring difficulty ratings, which presented higher precision. However, other investigators have presented similar results on their evaluation of the performance of certain NEI-VFQ items [35]. This suggests potential inherent validity issues related to the original instrument, rather than its translation and adaptation to the Greek language. Nonetheless, the important limitations of the scale detected with Rasch analysis may make it unsuitable for use.

Besides the aforementioned potential weakness of the instrument, certain limitations of our study may have to be considered. The translation process presented minor deviations from international recommendations. However, the research team is confident that these minor deviations had no actual impact on the translated outcome. On the other hand, the results are valid for the corresponding conditions of the patient groups and for the self-administered version of the instrument. Thus, prior to the usage of the instrument as a reliable index of VS-QoL in Greek patients with other diseases (i.e. diabetic retinopathy), further validation may be necessary in an appropriate sample of patients.

A series of vision-specific QoL studies conducted in homogenous populations have assessed the impact of systemic or ocular diseases on VS-QoL [1,2,36]. The outcomes of these studies could potentially modify the overall thinking on chronic disease management, since the patients' subjective perceptions regarding their visual impairment are not always in accordance with objective clinical parameters like the visual acuity or the visual fields. Literature suggests that in selective cases, non-clinical parameters such as educational background, financial state, or awareness regarding the disease have greater impact on the quality of life than objective clinical indices [37].

The majority of VS-QoL instruments attempt to quantify QoL by evaluating potential difficulties during a wide range of daily activities. It becomes obvious that the performance of QoL instruments (i.e. reliable assessment of subjective difficulty) depends heavily on the proper adaptation of the items to the cultural characteristics of the population studied, especially if the instrument was originally developed for a population with a different cultural background.

## Conclusion

Our results indicate that according to traditional validation methods, the Greek version of the NEI-VFQ 25 is a valid and reliable instrument for the assessment of VS-QoL in native populations. These findings are in agreement with the majority of previous research that translated and validated the instrument in series of ophthalmic patients by means of traditional validation methods, thus providing the theoretical framework for numerous VS-QoL studies. On the other hand, Rasch analysis revealed important misfits to the model, mainly of the "agreement" items, suggesting that the results of the corresponding subscales have to be interpreted with extreme caution. The significant limitations detected with Rasch analysis may render the instrument unsuitable for use. Further research is warranted for the re-evaluation of the performance of NEI-VFQ 25 as a VS-QoL instrument.

## Competing interests

The author(s) declare that they have no competing interests.

## Authors' contributions

GL conceived of the study and participated in its design and coordination. He also drafted the manuscript.

AK was involved in drafting the manuscript and revising it critically.

MP participated in data acquisition and study coordination.

MF performed the statistical analysis.

TT was involved in data acquisition and interpretation.

ET contributed to the study design and reviewed the manuscript critically.

DC coordinated the study and revised the manuscript.

All authors read and approved the final manuscript.

## Pre-publication history

The pre-publication history for this paper can be accessed here:



## Supplementary Material

Additional file 1 Multitrait-multimethod matrix. Matrix of correlation for the assessment of construct validity of sets of measures. Instead of "1" along the diagonal (as in a typical correlation matrix) we have substituted an estimate of the test-retest reliability of each measure. Click here for file
